# Human Differentiated Adipocytes as Surrogate Mature Adipocytes for Adipocyte-Derived Extracellular Vesicle Analysis

**DOI:** 10.3390/cells14110757

**Published:** 2025-05-22

**Authors:** Mangesh Dattu Hade, Bradley L. Butsch, Paola Loreto Palacio, Kim Truc Nguyen, Dharti Shantaram, Sabrena F. Noria, Stacy A. Brethauer, Bradley J. Needleman, Willa Hsueh, Eduardo Reátegui, Setty M. Magaña

**Affiliations:** 1 Department of Pediatrics, Center for Clinical and Translational Research, Nationwide Children’s Hospital, Columbus, OH 43202, USA; mangeshdattu.hade@nationwidechildrens.org (M.D.H.); bradleybutsch@gmail.com (B.L.B.); paolaloreto1703@gmail.com (P.L.P.); 2 Department of Chemical and Biomedical Engineering, The Ohio State University, Columbus, OH 43210, USA; nguyenkim.1@osu.edu (K.T.N.); reategui.8@osu.edu (E.R.); 3 Diabetes and Metabolism Research Center, The Ohio State University, Columbus, OH 43210, USA; dharti.shantaram@osumc.edu (D.S.); willa.hsueh@osumc.edu (W.H.); 4 Center for Minimally Invasive Surgery, Department of Surgery, Division of General and GI Surgery, The Ohio State University Wexner Medical Center, Columbus, OH 43210, USA; sabrena.noria@osumc.edu (S.F.N.); stacy.brethauer@osumc.edu (S.A.B.); bradley.needleman@osumc.edu (B.J.N.)

**Keywords:** extracellular vesicles, adipocyte, adipose, adipocyte-derived extracellular vesicles, obesity

## Abstract

Obesity is a growing global health concern, contributing to diseases such as cancer, autoimmune disorders, and neurodegenerative conditions. Adipose tissue dysfunction, characterized by abnormal adipokine secretion and chronic inflammation, plays a key role in these conditions. Adipose-derived extracellular vesicles (ADEVs) have emerged as critical mediators in obesity-related diseases. However, the study of mature adipocyte-derived EVs (mAdipo-EVs) is limited due to the short lifespan of mature adipocytes in culture, low EV yields, and the low abundance of these EV subpopulations in the circulation. Additionally, most studies rely on rodent models, which have differences in adipose tissue biology compared to humans. To overcome these challenges, we developed a standardized approach for differentiating human preadipocytes (preAdipos) into mature differentiated adipocytes (difAdipos), which produce high-yield, human adipocyte EVs (Adipo-EVs). Using visceral adipose tissue from bariatric surgical patients, we isolated the stromal vascular fraction (SVF) and differentiated preAdipos into difAdipos. Brightfield microscopy revealed that difAdipos exhibited morphological characteristics comparable to mature adipocytes (mAdipos) directly isolated from visceral adipose tissue, confirming their structural similarity. Additionally, qPCR analysis demonstrated decreased preadipocyte markers and increased mature adipocyte markers, further validating successful differentiation. Functionally, difAdipos exhibited lipolytic activity comparable to mAdipos, supporting their functional resemblance to native adipocytes. We then isolated preAdipo-EVs and difAdipo-EVs using tangential flow filtration and characterized them using bulk and single EV analysis. DifAdipo-EVs displayed classical EV and adipocyte-specific markers, with significant differences in biomarker expression compared to preAdipo-EVs. These findings demonstrate that difAdipos serve as a reliable surrogate for mature adipocytes, offering a consistent and scalable source of adipocyte-derived EVs for studying obesity and its associated disorders.

## 1. Introduction

The widespread prevalence of obesity has escalated into a formidable global health emergency, placing significant burdens on public health systems worldwide [1,2]. In 2019, an estimated 5 million obesity-related deaths occurred worldwide [3]. Obesity is intricately linked to various metabolic disorders, including type 2 diabetes mellitus, cardiovascular diseases, and non-alcoholic fatty liver disease [4,5,6]. Moreover, obesity is also implicated in the pathogenesis of several cancers, autoimmune diseases, and neurodegenerative disorders [7,8]. The complex interplay between obesity and these diseases underscores the multifaceted role of adipose tissue in maintaining metabolic homeostasis and overall health [9,10]. Adipose tissue, a dynamic endocrine organ, significantly influences energy balance, metabolic regulation, and immune responses [11]. Dysfunctional adipose tissue, characterized by altered adipokine secretion, chronic inflammation, and impaired adipocyte differentiation, is a hallmark of obesity and a crucial factor in the emergence of obesity-related comorbidities [12,13].

Recent research advances have illuminated the pivotal role of extracellular vesicles (EVs) in mediating intercellular communication and regulating metabolic processes [14,15,16]. EVs are small, membrane-bound particles secreted by cells that transport bioactive molecules (e.g., proteins, lipids, and nucleic acids) to recipient cells, thereby altering recipient cell genotypic and phenotypic function [14,15,17,18].

Mounting evidence indicates that obesity is associated with the enhanced production of adipose-derived EVs (ADEVs), which are instrumental in the pathogenesis of obesity and its related metabolic complications [19,20,21]. ADEVs are implicated in various physiological and pathological processes, including inflammation, insulin resistance, and lipid metabolism [21,22].

Despite the growing interest in studying ADEVs, there is a dearth of human ADEV studies, partly due to the technical and biological limitations of obtaining EVs from mature human adipocytes (mAdipo-EVs). mAdipos exhibit short viability in culture, produce low yields of EVs, and are difficult to handle due to their nonadherent properties [23,24,25]. Preadipocytes differentiated in vitro may also fail to fully replicate the biophysical and functional characteristics of adipocytes matured in vivo, particularly in terms of lipid storage, signaling, and extracellular vesicle composition. To address these limitations, and with the end goal of developing a robust human translational paradigm for isolating mature adipocyte-derived EVs, we first developed a surrogate mAdipo model by differentiating human preAdipos into mature-like difAdipos with functional, genotypical, and phenotypical similarities to mature adipocytes. Secondly, we used the validated difApos as a reliable and surrogate source of human adipocyte EVs, demonstrating classic EV and adipose markers.

In summary, our study provides a reproducible and reliable source of human mature-like adipose EVs. By leveraging human-based models, we aim to provide a more relevant understanding of the role of adipocyte-derived EVs in obesity-related disorders, thus addressing the critical need for human-based obesity EV paradigms [26].

## 2. Materials and Methods

### 2.1. Study Participants

This study complies with all relevant ethical guidelines. The Ohio State University Institutional Review Board (IRB# 2014H0471) approved the study, and all participants provided written informed consent. Visceral adipose tissue (VAT) samples were collected from 11 patients undergoing elective bariatric surgery at The Ohio State University (OSU) Center for Minimally Invasive Surgery. The mean age of participants was 41.6 ± 13 years, and the mean BMI was 41 ± 6. Clinical data are summarized in Table S1.

### 2.2. Adipose Tissue Dissociation and Mature Adipocyte Culture for EV Isolation

Adipose tissue samples were collected in the operating room during bariatric surgery and processed within 1 h using collagenase digestion, as previously described [27]. The cell suspension was centrifuged at 500× *g* for 10 min to generate three distinct phases (mature adipocytes, the stromal vascular fraction (SVF), and the SVF secretome). Floating mature adipocytes were carefully removed from the top layer and kept for culture. The supernatant was then carefully aspirated to avoid disturbing the SVF pellet, which was resuspended in sorting media for the preadipocyte isolation and differentiation protocol (see below). Mature adipocytes were cultured for 24 h in Dulbecco’s Modified Eagle Medium/Ham’s Nutrient Mixture F-12 (DMEM/F12), supplemented with 40% M199 basal medium with Earle’s salts and L-glutamine (MCDB201), 2% fetal bovine serum (FBS), 1× penicillin/streptomycin, 1 nM dexamethasone, 0.1 mM L-ascorbic acid 2-phosphate (LAAP), 1× Insulin-Transferrin-Selenium (ITS) Mix, 1× linoleic acid-albumin, 5 μg/μL insulin, and 1 nM triiodothyronine (T3).

### 2.3. Preadipocyte (preAdipo) Isolation and Differentiation into Differentiated Mature Adipocytes (difAdipos)

The resuspended SVF pellet was filtered through a 100 µm cell strainer into fresh tubes, repeating the washing step to ensure the thorough isolation of SVF cells. Following another round of centrifugation (at 1200 rpm for 10 min at RT or 4 °C) and aspiration of the supernatant, the SVF pellet was treated with ACK lysis buffer on ice for three minutes to lyse any remaining red blood cells, followed by dilution and filtration through a 40 µm cell strainer. Finally, the SVF pellet containing cells was resuspended in sorting medium and seeded into culture plates until the cells reached approximately 80% confluency. The differentiation process commenced with a 24 h incubation in growth medium, designated as Day 0. Throughout the differentiation period, the medium was replaced every 48 h. Cells were maintained for up to 28 days in adipogenic induction medium 1 and 2 (AIM1 and AIM2). For media composition, see Table S3.

### 2.4. Cell Line and Culture

The human embryonic kidney epithelial cell line HEK-293T (ATCC^®^ CRL-3216™) was purchased from the American Type Culture Collection (ATCC, Manassas, VA, USA). Cells at passage number 14 were cultured in Dulbecco’s Modified Eagle Medium (DMEM; Gibco, Grand Island, NY, USA) supplemented with 10% fetal bovine serum (FBS; Gibco, Grand Island, NY, USA) and 1% penicillin–streptomycin (Solarbio, Beijing, China). Cultures were maintained in standard tissue culture dishes at 37 °C in a humidified atmosphere containing 5% CO_2_.

### 2.5. ADEV Isolation via Tangential Flow Filtration (TFF)

Culture media from preAdipo (10 mL), difAdipo (9 mL), and mAdipo (9 mL) were first filtered through 0.2 μm filters and concentrated to a final volume of 5 mL. The concentrated media underwent diafiltration using tangential flow filtration (TFF) with a 500 kDa filter at a flow rate of 35 mL/min, as described previously [27]. After TFF, the retentates were further concentrated to 100 μL using 30 kDa molecular weight cutoff (MWCO) centrifugal filter units (MilliporeSigma Amicon Ultra, Burlington, MA, USA) by centrifugation at 4000× *g* for 30 min. Briefly, the media were first passed through 0.2 μm PES syringe filters (MilliporeSigma, Burlington, MA, USA) to remove cells and debris. The filtered media were concentrated to a volume of 5 mL, then adjusted to a total volume of 7 mL using sterile PBS prior to tangential flow filtration (TFF). The TFF was performed using a self-assembled system equipped with a 500 kDa molecular weight cutoff (MWCO) hollow fiber filter (Repligen, Waltham, MA, USA). A peristaltic pump (Cole-Parmer, Vernon Hills, IL, USA) circulated the media at a constant flow rate of 35 mL/min. During diafiltration, phosphate-buffered saline (PBS, pH 7.4) was added to the reservoir at the same rate as the permeate removal to maintain a constant volume and facilitate the removal of low-molecular-weight proteins, free nucleic acids, and other soluble contaminants. The 7 mL starting volume was selected to account for 2 mL of dead volume in the product container and 5 mL of liquid within the tubing, thereby protecting EVs from desiccation and enabling consistent diafiltration.

Following TFF, the enriched EV fraction (retentate) was collected (~2 mL) and further concentrated using 30 kDa MWCO centrifugal filter (MilliporeSigma Amicon Ultra, Burlington, MA, USA) by centrifugation at 4000× *g* for 30 min at 4 °C. The final EV samples were resuspended in 100 μL of sterile PBS for downstream characterization and analysis.

### 2.6. Microfluidic Resistive Pulse Sensing (MRPS)

The concentration and size distribution of particles were assessed using microfluidic resistive pulse sensing (MRPS) on the Spectradyne nCS1 instrument (Spectradyne, Torrance, CA, USA). Measurements were conducted with C-400 polydimethylsiloxane cartridges, enabling detection within an approximate size range of 65–400 nm [27]. Data processing and analysis were performed using the nCS1 Data Analyzer software (Spectradyne, Torrance, CA, USA).

### 2.7. Western Blotting

EV samples were lysed using radioimmunoprecipitation assay (RIPA) buffer (Thermo Scientific, Waltham, MA, USA) supplemented with protease and phosphatase inhibitors (Thermo Scientific, Waltham, MA, USA) and incubated on ice for 15 min. Protein concentration was measured with the Micro BCA™ Protein Assay Kit (Thermo Scientific, Waltham, MA, USA). Equal protein amounts were mixed with Laemmli buffer containing 2-mercaptoethanol (Sigma-Aldrich, St. Louis, MO, USA) and separated via SDS-PAGE on 4–20% Mini-PROTEAN^®^ TGX Stain-Free gels (Bio-Rad, Hercules, CA, USA). The proteins were then transferred onto polyvinylidene fluoride (PVDF) membranes (Bio-Rad, Hercules, CA, USA). After blocking, membranes were incubated overnight at 4 °C with primary antibodies prepared in TBS-T, followed by a 1 h incubation with horseradish peroxidase-conjugated secondary antibodies at room temperature. Protein detection was carried out using enhanced chemiluminescence (ECL) reagents (Bio-Rad, Hercules, CA, USA), and signals were visualized using the Bio-Rad ChemiDoc™ MP imaging system.

### 2.8. Quantification of Real-Time Polymerase Chain Reaction (qRT-PCR)

Total RNA was extracted using the RNeasy Mini Kit (Qiagen, Germantown, MD, USA) following the manufacturer’s protocol and quantified with a NanoDrop spectrophotometer (Thermo Scientific, Waltham, MA, USA). The purified RNA was then reverse-transcribed into complementary DNA (cDNA) using the High-Capacity cDNA Reverse Transcription Kit (Applied Biosystems, Foster City, CA, USA). Quantitative real-time PCR was conducted using PowerUp™ SYBR™ Green Master Mix (Thermo Fisher, Waltham, MA, USA) on an Applied Biosystems real-time PCR system. The forward and reverse primer sequences are provided in Table S4. β-actin served as the internal reference gene for normalization.

### 2.9. Lipolysis Assay

To investigate mature adipocyte functional activity, a lipolysis assay was performed using the Lipolysis Assay Kit (Abcam, Cambridge, MA, USA) per the manufacturer’s instructions. Day 0 (preAdipos), Day 28 (difAdipos), and cultured HEK293 (negative control) cells were washed twice with Lipolysis Wash Buffer, then stimulated with 100 nM isoproterenol for 4 h. Absorbance at 570 nm was measured using the SpectraMax iD3 (Molecular Devices, San Jose, CA, USA), and glycerol concentration was quantified using a standard curve.

### 2.10. Transmission Electron Microscopy (TEM)

TEM was performed as previously described [28]. Briefly, TEM grids were first treated with plasma for high surface EV absorption. Subsequently, 10 µL of EV samples were drop-casted onto the plasma-treated surface. The samples were incubated for 1 min and then blotted with filter paper to remove excess liquid. The TEM grids were washed twice in DI water and stained with UranyLess EM contrast stain (Electron Microscopy Science, Hatfield, PA, USA) for 22 s. The TEM grids were dried overnight prior to imaging. TEM images were obtained using Tecnai TF-20 microscope under a 200 kV, bright-field imaging mode.

### 2.11. Single EV Analysis Using Total Internal Reflection Fluorescence Microscopy (TIRF)

The isolated EVs were captured and analyzed on a gold functionalized biochip following our previous protocol [27]. Briefly, the EVs collected on Day 2 (preAdipo-EVs) and Day 28 (difAdipo-EVs) were permeabilized and hybridized with molecular beacons (Table S5) in 0.5× Tris EDTA (TE) buffer at 0.2 µM concentration for 2 h at 37 °C in a dark environment to facilitate molecular beacon hybridization to the target RNA. The EV samples were incubated on the biochip surface and captured by functionalized anti-CD63 and anti-CD9 antibodies. Proteins were detected using fluorescent-dye-conjugated antibodies. Finally, a 10 × 10 array of images was acquired via total internal reflection fluorescence microscope (TIRFM; Nikon, Melville, NY, USA) for each well. Relative and total fluorescence intensities of the sample were obtained from custom-built algorithms that were previously reported [29,30].

- Antibody functionalization of the gold-coated biochip:

As previously described in our earlier work, gold-coated biochips were prepared and functionalized using biotin-PEG-SH94. Each well received 20 μL of solution, with all incubation steps performed on a shaker to ensure consistent reagent distribution. Prior to functionalization, each well was thoroughly rinsed by pipetting deionized (DI) water up and down ten times. For NeutrAvidin (NA) immobilization, a 50 μg/mL solution of NA (Thermo Fisher Scientific, Waltham, MA, USA) in phosphate-buffered saline (PBS) was added to each well and incubated for 1 h at room temperature to allow for specific binding to the biotin groups on the chip surface. Excess NA was removed with three sequential washes using PBS, pipetted ten times per rinse.

- Immobilization of biotinylated capture antibodies:

Capture antibodies were biotinylated using the EZ-Link Sulfo-NHS-LC-Biotin kit (Thermo Fisher Scientific) according to the manufacturer’s instructions. The biotinylated antibodies were then diluted to 10 μg/mL in 1% (*w*/*v*) bovine serum albumin (BSA) prepared in PBS. This antibody solution was added to each well and incubated for 1 h at room temperature. After incubation, unbound antibodies were removed by washing with PBS (pipetted up and down ten times), repeated for a total of three rinses.

- Hybridization of molecular beacons (MBs):

Each molecular beacon (MB) was diluted to 5 μM in 12.5× Tris-EDTA (TE) buffer (Sigma-Aldrich) prepared with DI water, which also served to stabilize the MBs and gently permeabilize EV membranes for RNA access. The MB mixture was then diluted 1:25 in the sample and added to the biochip. Hybridization was carried out for 2 h at 37 °C in the dark to promote specific interaction with target RNA sequences.

- EV capture on the biochip surface:

To minimize nonspecific EV adhesion and maintain single-particle resolution, each well was pre-blocked with 3% (*w*/*v*) BSA in PBS for 1 h at room temperature. After removing BSA, biofluid samples were added and incubated for 2 h in the dark at room temperature. The wells were then rinsed with PBS for 5 min, followed by ten gentle pipetting cycles to remove unbound EVs and MBs. This washing step was repeated four times under low-light conditions.

- Detection of surface proteins using fluorophore-conjugated antibodies:

To block nonspecific interactions during the detection step, a 3% (*w*/*v*) BSA solution in PBS was first added to each well and incubated for 1 h at room temperature. After discarding the blocking solution, detection antibodies were prepared at 1 μg/mL in 10% (*w*/*v*) normal goat serum (NGS; Thermo Fisher Scientific, Waltham, MA, USA) and introduced into the wells. The wells were incubated for 1 h in the dark at room temperature. To remove unbound antibodies, PBS was added for a 5 min incubation, followed by ten pipetting cycles. This rinse was repeated three times in a dim environment. All experiments were performed using a minimum of three independent biological replicates (n = 3).

### 2.12. EV Labeling and Uptake Assay

EVs were labeled with MemGlow™ 488 (Cytoskeleton, Inc., Denver, CO, USA) according to the manufacturer’s instructions. Briefly, EVs were incubated with the dye in PBS for 30 min at room temperature in the dark, and excess dye was removed using a 10 kDa Amicon Ultra filter (Millipore, Burlington, MA, USA) with multiple PBS washes. HMC3 microglial cells, passage 6, and THP-1 monocytes, passage 7, were seeded in 24-well plates at a density of 1 × 10^5^ cells/well and incubated for 24 h. HMC3 cells were cultured in DMEM/F-12 medium (Gibco, Grand Island, NY, USA) supplemented with 10% fetal bovine serum (FBS) and 1% penicillin–streptomycin. THP-1 cells were maintained in RPMI-1640 medium (Gibco, Grand Island, NY, USA) with 10% FBS and 1% penicillin–streptomycin and were differentiated using phorbol 12-myristate 13-acetate (PMA, 100 ng/mL, Sigma-Aldrich, St. Louis, MO, USA) for 24 h prior to EV treatment. Cells were then treated with 1 × 10^9^ MemGlow488-labeled EVs per well and incubated for 24 h at 37 °C. PBS-only treatment was used as a control. Following incubation, cells were washed with PBS and fixed with 4% paraformaldehyde for 15 min at room temperature. Cells were then stained with NucBlue™ Live ReadyProbes™ Reagent (Invitrogen, Carlsbad, CA, USA) for 30 min at room temperature, followed by three PBS washes. EV uptake was visualized using a Keyence BZ-X800 fluorescence microscope (Keyence Corporation, Itasca, IL, USA) with filters for MemGlow488 and DAPI, and images were analyzed using BZ-X800 Analyzer software.

### 2.13. Statistical Analysis

All data analyses were performed using GraphPad Prism software (version 10). Experimental results are presented as mean ± standard deviation (SD), with a minimum of three independent biological replicates (n = 3) per group, unless otherwise noted. Statistical significance was assessed at a threshold of *p* < 0.05.

For multi-group comparisons, ordinary one-way ANOVA followed by multiple comparisons tests was used. For direct comparisons between two groups, a Student’s *t*-test was applied. Specific tests and sample sizes are indicated in the corresponding figure legends.

## 3. Results

### 3.1. Multiparametric Isolation and Characterization of ADEVs

**Figure 1:**
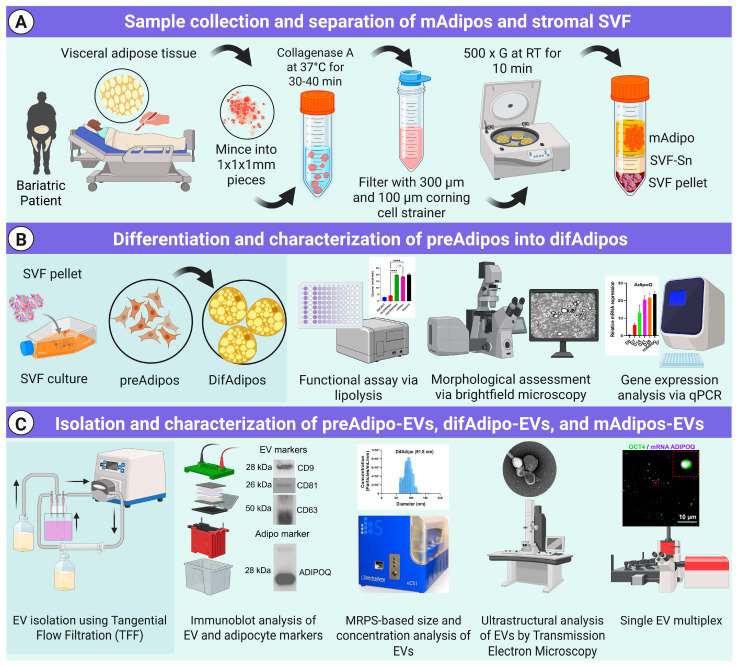
Workflow for the isolation, differentiation, and characterization of human preadipocytes, differentiated adipocytes, and their EVs. (**A**) Visceral adipose tissue obtained from bariatric patients was enzymatically digested using Collagenase A at 37 °C for 30–40 min, followed by filtration through 300 µm and 100 µm cell strainers. Subsequent centrifugation (500× *g*, RT, 10 min) yielded three distinct fractions: stromal vascular fraction (SVF), SVF supernatant (SVF-SN), and mature adipocytes (mAdipos). The SVF-derived preadipocytes (preAdipos) were cultured and induced to differentiate into differentiated mature adipocytes (difAdipos) over 28 days. Conditioned media from adipocyte cultures were collected and processed using tangential flow filtration (TFF) for EV isolation. (**B**) Characterization of preAdipos, difAdipos, and mAdipos included functional assays assessing lipolysis, morphological analysis via brightfield microscopy, and gene expression analysis using quantitative PCR. (**C**) Isolated EVs from preAdipos (preAdipo-EVs) and difAdipos (difAdipo-EVs) underwent extensive characterization, including immunoblot analysis of EV and adipocyte markers (CD9, CD81, CD63, and ADIPOQ), size distribution and concentration analysis via microfluidic resistive pulse sensing (MRPS), ultrastructural evaluation using transmission electron microscopy (TEM), and single-EV multiplexing analysis. Extracellular vesicles (EVs); stromal vascular fraction (SVF); SVF supernatant (SVF-SN); mature adipocytes (mAdipos); preadipocytes (preAdipos); differentiated mature adipocytes (difAdipos); microfluidic resistive pulse sensing (MRPS); transmission electron microscopy (TEM); (**** *p* < 0.0001); ns, not significant.

We have developed a comprehensive pipeline for isolating and characterizing EVs from various human adipose tissue biospecimens [27] (Figure 1, Table S2). While most human EV biomarker studies have focused on biofluids or in vitro EV sources, isolating tissue-derived EVs presents technical challenges that must be addressed to maintain the integrity of both the cells and the EVs [31]. To ensure our isolation method produced intact adipocyte-derived EVs, we performed extensive bulk and single EV characterization, as detailed below in Figure 1.

### 3.2. Visceral Adipose-Derived Preadipocytes Serve as a Surrogate Source for Mature Adipocytes

**Figure 2:**
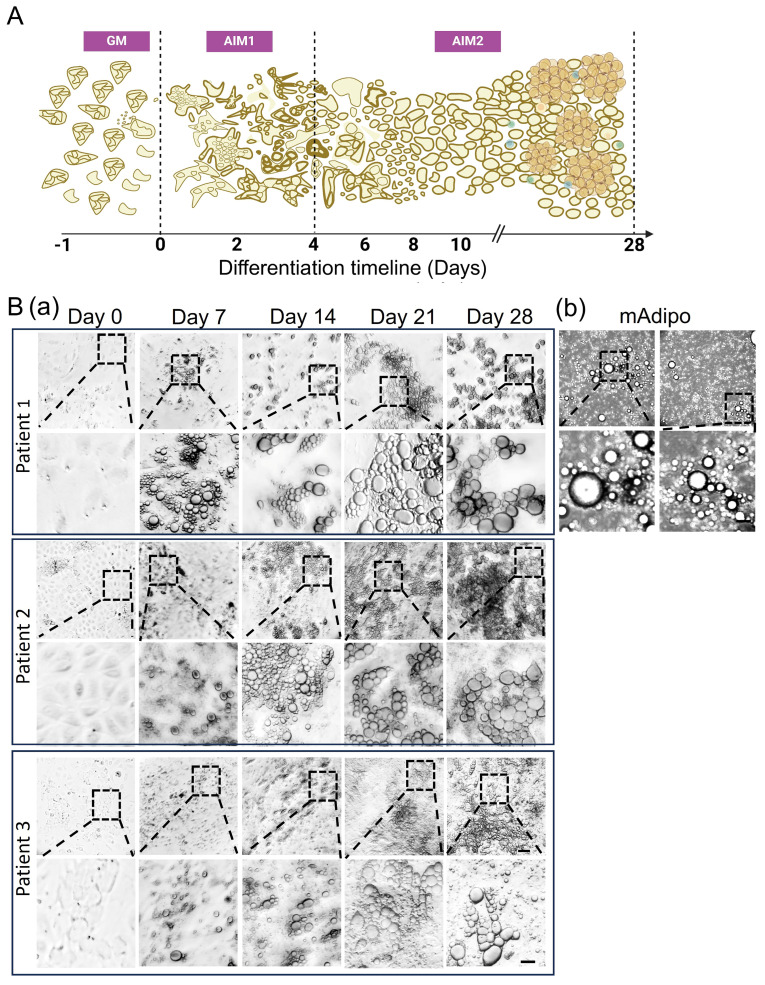
Human preadipocytes undergo differentiation and acquire a mature adipocyte phenotype. (**A**) Differentiation timeline: At Day 1, cells reached ~80% confluency in growth medium (GM). Differentiation was initiated on Day 0 following a 24 h incubation in GM by replacing the medium with adipogenic induction medium 1 (AIM1). The transition to adipogenic induction medium 2 (AIM2) was performed to facilitate further maturation, leading to the acquisition of a differentiated mature adipocyte (difAdipo) phenotype by Day 28. (**B**) Morphological changes during adipogenesis: Preadipocytes were sourced from three independent bariatric surgical patients. Morphological changes were documented every seven days for each patient. (**a**) Representative brightfield microscopy images illustrate the progressive morphological changes occurring during preadipocyte differentiation into difAdipos. Lipid droplet accumulation becomes progressively evident throughout the differentiation process. (**b**) The morphology of difAdipos at Day 28 closely resembles that of primary mature adipocytes (mAdipos), which were directly isolated from patient-derived visceral adipose tissue and maintained in culture for 24 h. Images are representative of three independent biological replicates (n = 3). Dashed boxes highlight selected regions, shown as enlarged, high-magnification images in subsequent panels to provide detailed visualization of cellular morphology. Scale bar = 50 µm for main images; 10 µm for enlarged high-magnification images. GM, growth medium; AIM1, adipogenic induction medium 1; AIM2, adipogenic induction medium 2; difAdipo, differentiated mature adipocyte; mAdipos, mature adipocytes.

We established a human-based protocol for isolating, differentiating, and characterizing SVF-derived preadipocytes that yielded a reliable surrogate source of differentiated mature adipocytes (difAdipos) (see Materials and Methods). Preadipocytes exhibited significant morphological changes during the differentiation process, accumulating larger lipid droplets and developing a mature phenotype, as confirmed by brightfield imaging (Figure 2B).

### 3.3. Differential Expression of Preadipocyte and Mature Adipocyte Markers During Adipocyte Differentiation

**Figure 3:**
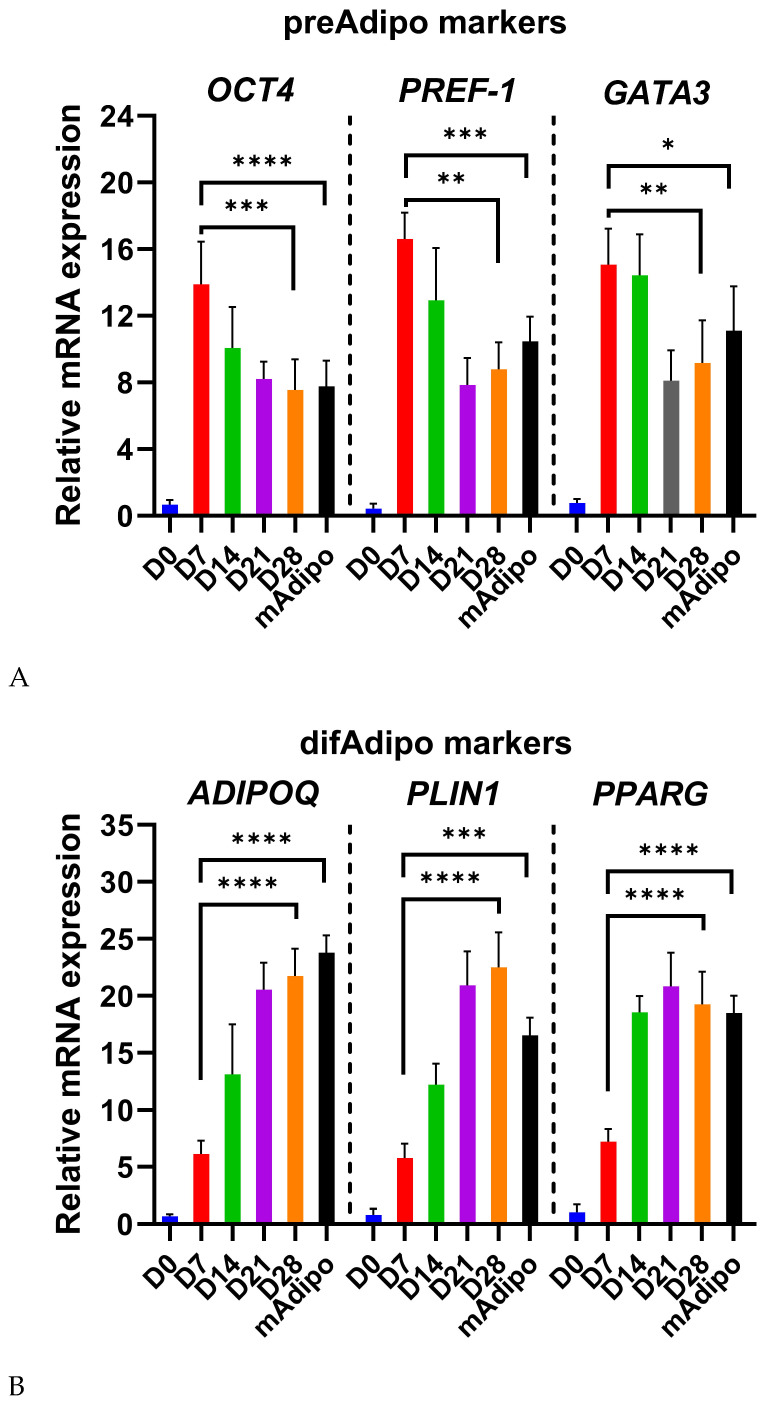
Transcript profile in preadipocytes, differentiated adipocytes, and mature adipocytes. (**A**) Relative mRNA expression levels of preadipocyte markers (*OCT4*, *PREF1*, *GATA3*) and (**B**) mature adipocyte markers (*ADIPOQ*, *PLIN1*, *PPARG*) were measured at Day 0 (D0), D7, D14, D21, D28, and in terminally differentiated mAdipos. *OCT4* expression peaked at D7 and decreased thereafter, indicating early differentiation. PREF-1 expression increased to a peak at D7 and decreased towards D28, consistent with its inhibitory role in adipogenesis. *GATA3* expression peaked at D7 and D14 and remained elevated in mAdipos, indicating its involvement in early differentiation. Adipocyte markers *ADIPOQ*, *PLIN1*, and PPARG exhibited a progressive increase, peaking at D28 and in mAdipos, reflecting mature adipocyte function (n = 3 independent biological replicates). The statistical analysis was performed using ordinary one-way ANOVA with Bartlett’s test. *OCT4*, octamer-binding transcription factor 4; *PREF-1*, preadipocyte factor 1; *GATA3*, GATA-binding protein 3; *ADIPOQ*, adiponectin; *PLIN*, perilipin; *PPARG*, peroxisome proliferator-activated receptor gamma. A value of *p* < 0.05 was deemed statistically significant (* *p* < 0.05; ** *p* < 0.01; *** *p* < 0.001; **** *p* < 0.0001).

We next investigated gene transcripts of known preAdipo markers (*OCT4, PREF-1*, and *GATA3*) and mature adipocyte markers (*ADIPOQ, PLIN1,* and *PPARG*) at different time points during adipocyte differentiation (Figure 3). We hypothesized that difAdipos would acquire a genetic program similar to mAdipos and lose the undifferentiated transcript profile that characterizes preAdipos. As expected, *OCT4* expression was low at Day 0 (D0), peaked at D7 (*p* < 0.001), and decreased as differentiation progressed and cells committed adipogenesis (Figure 3A).

We saw a similar pattern in *PREF-1* expression levels. *PREF-1* is an inhibitor of adipogenesis, and it is downregulated as cells undergo differentiation. Consistent with this observation, preAdipo *PREF-1* levels were relatively low at D0, peaked at D7 (*p* < 0.0001), and were decreased by D28 and in mAdipos (Figure 3A). In contrast to *OCT4* and *PREF-1, GATA3* levels persisted in both the early and intermediate differentiation stages. *GATA3* expression peaked at D7 (*p* < 0.01) and D14, decreased through D21 and D28, but remained elevated in mAdipos compared to D28 (Figure 3A). This expression pattern is consistent with studies showing *GATA3’s* involvement in regulating adipogenesis.

Concurrently, the expression of the mature adipocyte markers *ADIPOQ, PLIN1*, and *PPARG* showed a clear increasing trend during differentiation, as previously reported. *ADIPOQ* expression was low at D0 and D7, increased steadily from D14 onwards, and peaked at D28 (*p* < 0.0001), with levels comparable to those seen in our mAdipos (Figure 3B). *PLIN1* plays a crucial role in regulating lipid storage and mobilization by protecting lipid droplets from lipolysis in adipocytes. We observed gradually increasing levels of *PLIN1* expression from D0 onward, significantly increasing at D21 and D28 (*p* < 0.0001), signifying lipid accumulation and adipocyte maturation. Similarly, *PPARG* expression progressively increased from D0 to D28, with the highest expression observed at D21 and D28 (*p* < 0.0001), and slightly lower expression in mAdipos, highlighting its crucial role as a regulator of adipogenesis (Figure 3B). In summary, the dynamic transcriptional program we observed reflects the successful differentiation of preadipocytes into difAdipos exhibiting mature adipocyte transcriptional profiles.

### 3.4. Differentiated Adipocytes Exhibit Lipolytic Function Comparable to Mature Adipocytes

**Figure 4:**
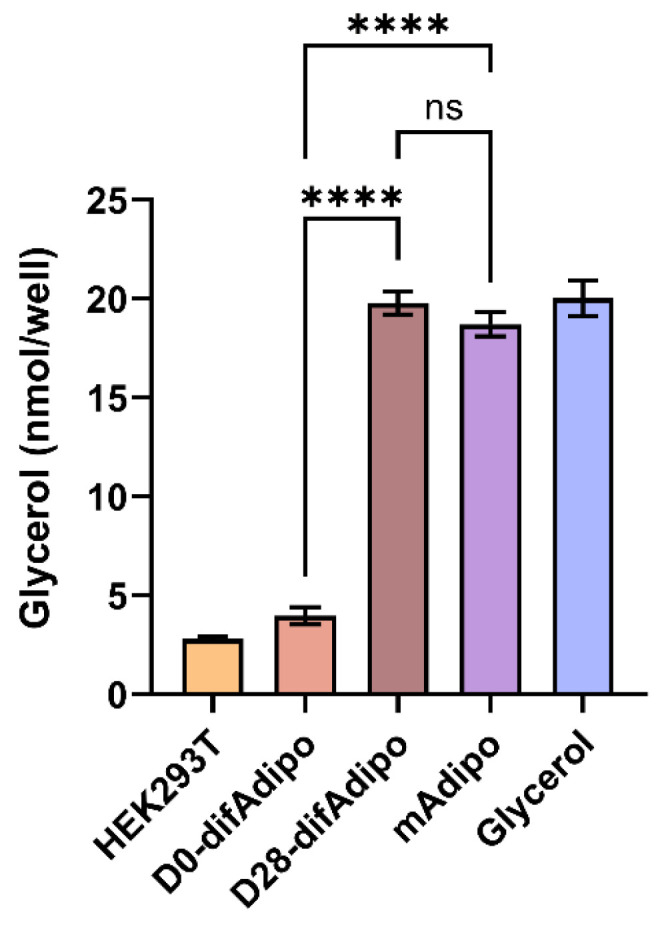
Differentiated adipocytes (difAdipos) exhibit lipolytic function comparable to mature adipocytes (mAdipos). Lipolytic activity was assessed in preAdipos, difAdipos, and mAdipos by stimulating cells with isoproterenol and measuring glycerol release. D0 difAdipos reflected undifferentiated adipocytes and exhibited low-level lipolytic activity similar to HEK293T cells (negative control). D28 difAdipos exhibited lipolytic activity comparable to mAdipos and glycerol (positive control), validating the differentiation protocol and confirming that difAdipos functionally resemble mature adipocytes in their lipolytic activity (n = 3 independent biological replicates). Statistical analysis was performed using one-way ANOVA with Sidak’s multiple comparison test. A value of *p* < 0.05 was deemed statistically significant (**** *p* < 0.0001); ns, not significant.

Further, we next sought to functionally validate the mAdipo phenotype of our difAdipos by investigating their ability to perform the prototypic mature adipocyte function of lipolysis. Cells were stimulated with the lipolytic agent isoproterenol, and lipolysis was assessed by quantifying the stimulated release of glycerol into the culture medium (Figure 4). We hypothesized that the lipolytic activity of difAdipos would not differ from that of mAdipos, suggesting that our difAdipos could serve as functional surrogates of mAdipos. Compared to undifferentiated D0 preadipocytes, D28 difAdipos achieved a comparable degree of lipolytic activity as mAdipos. These findings demonstrate our ability to generate human difAdipos that are not only morphologically and genotypically comparable to mAdipos but also functionally resemble mAdipos.

### 3.5. Multiparametric Characterization of EVs from Differentiated Adipocytes

**Figure 5:**
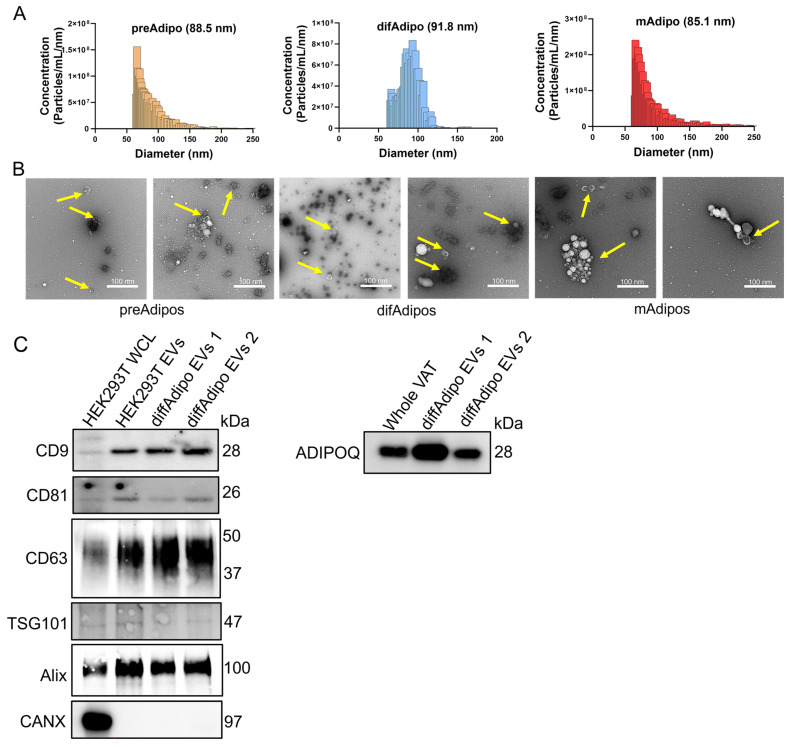
Multiparametric characterization of matched adipose tissue-derived EVs from the same bariatric patients. (**A**) MRPS analysis of EVs from preAdipos, difAdipos, and mAdipos from the same individuals (n = 3 independent biological replicates). (**B**) TEM imaging showed a polydispersed population of particles, including small EVs with a characteristic ‘cup-shaped’ morphology. Images are representative of EVs from preAdipo, difAdipo, and mAdipo groups, which exhibited similar ultrastructural features. Yellow arrows indicate individual EVs. (**C**) Immunoblot analysis of EVs from difAdipos reveals heterogeneous expression of typical EV (CD9, CD81, CD63, TSG101, and Alix), non-EV (CANX), and adipose-specific (ADIPOQ) markers. Full-size uncropped blot images for panel (**C**) are provided in Supplementary Figure S1. MRPS, microfluidic resistive pulse sensing; TEM, transmission electron microscopy.

After validating our difAdipos as robust surrogates for mAdipos, we next sought to characterize EVs from matched preAdipos, difAdipos, and mAdipos obtained from the same individual (Figure 5A). Microfluidic resistive pulse sensing analysis revealed a relatively narrow particle size distribution among the EVs from preAdipos, difAdipos, and mAdipos. Median particle diameters were similar across the three Adipo populations (88.5 nm for preAdipos, 91.8 nm for difAdipos, and 85.1 nm for mAdipos). All three ADEV sources had similar mean particle concentrations: 6.26 × 10^10^ particles/mL for mAdipos, 3.47 × 10^10^ particles/mL for preAdipos, and 2.33 × 10^10^ particles/mL for difAdipos (Figure 5A). These results suggest that our differentiation protocol did not alter the ability of difAdipos to generate small EVs that were similar in size and concentration to native populations (i.e., preAdipos and mAdipos), which did not undergo in vitro maturation.

TEM revealed a polydispersed and prominent population of small EVs with the characteristic artefactual ‘cup-shaped’ morphology (Figure 5B). Immunoblotting of difAdipo-EVs demonstrated a heterogeneous expression of the typical EV markers CD9, CD81, CD63, TSG101, Alix, and the absence of the non-EV marker calnexin (CANX) (Figure 5C and Figure S1). The adipocyte marker ADIPOQ was also detected (Figure 5C and Figure S1). Collectively, these findings highlight the diversity of tissue-derived EV populations from varied adipocyte sources within the same individual and at different stages of adipocyte differentiation.

### 3.6. High-Resolution, Simultaneous Detection of Human Adipose-Derived EV Proteins and RNA Using Total Internal Reflection Fluorescence (TIRF) Microscopy

**Figure 6:**
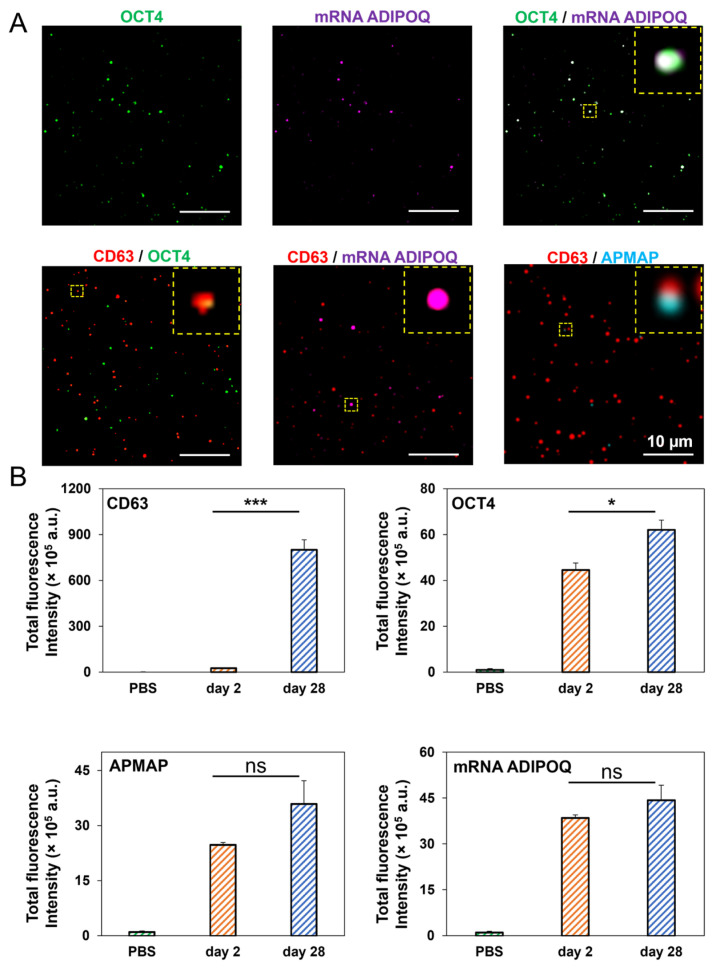
Single EV multiparametric characterization comparing preAdipo-EVs (Day 2) and difAdipo-EVs (Day 28). (**A**) Representative TIRFM images illustrating difAdio-EVs were captured by anti-CD9 and anti-CD63 antibodies and immobilized on a biochip surface. Detection antibodies against CD63, OCT4, and APMAP were used to identify EV surface proteins. A fluorescence-labeled molecular beacon detected the mRNA *ADIPOQ* within those EVs. The merged image reveals difAdipo-EVs defined by colocalization of OCT4 and mRNA *ADIPOQ*, CD63 and OCT4, CD63 and mRNA *ADIPOQ*, and CD63 and APMAP; scale 10 µm. (**B**) The relative fluorescence intensity of EVs collected at Day 2 and Day 28 was analyzed for the tetraspanin marker CD63, the undifferentiated cell marker OCT4, the adipocyte plasma membrane-associated protein APMAP, and the adipose tissue gene marker mRNA *ADIPOQ*. OCT4, octamer-binding transcription factor 4; ADIPOQ, adiponectin; APMAP, astrocyte membrane-associated protein (n = 3 independent biological replicates). Statistical analysis was performed using Student’s *t*-test. A value of *p* < 0.05 was deemed statistically significant (* *p* < 0.05; *** *p* < 0.001); ns, not significant.

TIRF microscopy was utilized to achieve simultaneous detection of human adipose-derived EV proteins and RNA and colocalization of the same (Figure 6A,B). First, we investigated the colocalization of EV surface proteins and EV mRNA cargo in preAdipo-EVs and difAdipo-EVs to confirm the specificity of adipocyte-EVs. DifAdipo-EVs revealed high colocalization between OCT4 protein and *ADIPOQ* mRNA, confirming the adipocyte specificity of the EVs. The tetraspanin marker CD63 showed less to no colocalization with other biomarkers (Figure 6A), underscoring the heterogeneous expression profile of tetraspanins.

Next, we quantified the relative fluorescence intensity of EV surface markers and mRNA cargo (Figure 6B). DifAdipo-EVs collected on Day 28, compared to preAdipo-EVs collected on Day 2, expressed a significantly higher level of the canonical EV marker CD63 (*** *p* < 0.001). Interestingly, the protein marker for undifferentiated adipocytes, OCT4, was also significantly higher on difAdipo-EVs than preAdipo-EVs (* *p* < 0.05). The expression levels of adipose tissue-specific protein and gene markers, such as adipocyte plasma membrane-associated protein (APMAP) and ADIPOQ, were similar between the difAdipo-EVs and preAdipo-EVs. In summary, APMAP and ADIPOQ exhibited stable expression levels across the maturation process, while CD63 and OCT4 were dynamically regulated during the same differentiation process.

### 3.7. Efficient Internalization of DifAdipo- and MAdipo-Derived EVs by HMC3 Microglia and THP-1 Macrophages

**Figure 7:**
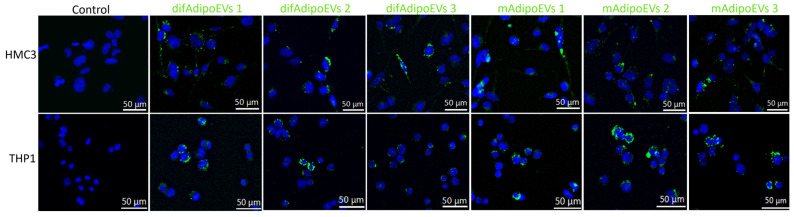
Uptake of difAdipo- and mAdipo-EVs by HMC3 microglia and THP-1 macrophages. HMC3 microglia and PMA-differentiated THP-1 macrophages were incubated for 24 h at 37 °C with PBS (control), MemGlow™ 488-labeled difAdipo-EVs or MemGlow™ 488-labeled mAdipo-EVs (1 × 10^9^ EVs/well). EVs appear in green; nuclei were counterstained with Hoechst 33342 (NucBlue™, Invitrogen; blue). Representative fields from three independent biological replicates (n = 3). Scale bars, 50 μm.

To explore whether difAdipo-EVs and mAdipo-EVs can engage recipient cells in a functionally meaningful way, we first assessed their cellular internalization. HMC3 microglia and PMA-differentiated THP-1 macrophages were incubated with MemGlow™ 488-labeled difAdipo-EVs or mAdipo-EVs at 1 × 10^9^ EVs per well for 24 h at 37 °C, with PBS-only as a negative control (Figure 7). Fluorescence imaging revealed that both EV populations were taken up robustly by each cell type in all three biological replicates. In HMC3 cells, difAdipo-EVs localized as intense perinuclear puncta, whereas mAdipo-EVs showed a more diffuse cytosolic distribution; THP-1 macrophages exhibited mixed clustered and diffuse patterns for both EV types. These results establish that difAdipo- and mAdipo-derived EVs are functionally competent for uptake in immune-relevant cells, laying the groundwork for downstream assays of macrophage polarization and hepatocyte function.

## 4. Discussion

Adipose-derived EVs are increasingly recognized as critical orchestrators in obesity-associated diseases [32]. Limited studies have characterized human adipocyte EVs [33,34,35,36,37]; however, no study has systematically studied paired difAdipo-EVs and mAdipo-EVs at bulk and single EV resolutions. Herein, we present a reproducible and scalable surrogate source of human mature-like adipocytes (difAdipos) that exhibit phenotypic, genotypic, and functional properties paralleling those of paired mature adipocytes (mAdipos). Furthermore, we demonstrate that in vitro difAdipos can release ADEVs comparable to ADEVs released by mature adipocytes. Finally, we provide the first high-resolution, single EV analysis of human difAdipo-EVs, demonstrating simultaneous adipocyte-enriched gene transcript and surface protein markers.

Underpinning these functional and EV-based findings, we investigated the temporal expression profiles of classic preadipocyte and adipocyte markers [38,39,40,41]. The coordinated temporal regulation of both preadipocyte and mature adipocyte markers during differentiation provides strong evidence that our difAdipo model faithfully recapitulates key aspects of human adipogenesis. The early peak in *OCT4* and *PREF-1* transcript levels at Day 7, followed by their subsequent decline, is consistent with their roles in maintaining progenitor identity and inhibiting differentiation, respectively [42,43]. The sustained expression of *GATA3* through intermediate time points further underscores its involvement in orchestrating the commitment phase of adipogenesis [38]. In parallel, the steady upregulation of canonical adipocyte markers—*ADIPOQ*, *PLIN1*, and *PPARG* from Day 14 onward mirrors the acquisition of lipid-handling and endocrine functions characteristic of mature adipocytes [44,45,46]. Notably, the convergence of *ADIPOQ* levels in difAdipos and primary mAdipos by Day 28 confirms that our protocol drives not only morphological but also transcriptional maturation [47]. Together, these expression dynamics validate our differentiation strategy and establish a robust genetic framework upon which to interpret subsequent functional and EV-based studies. The effectiveness of our differentiation protocol is supported not only by phenotypic, transcriptional, and functional validation but also by the inclusion of a rationally designed adipogenic induction medium. AIM1 and AIM2 incorporate insulin, dexamethasone, IBMX, indomethacin, triiodothyronine (T3), and supportive factors such as linoleic acid-albumin and L-ascorbic acid 2-phosphate. These compounds act synergistically to activate key transcriptional regulators (e.g., PPARγ, C/EBPs), promote lipid accumulation, and enhance extracellular matrix remodeling during differentiation [39,48,49,50,51]. Briefly, dexamethasone and IBMX initiate early adipogenic signaling, while insulin and T3 support metabolic maturation. Indomethacin acts as a PPARγ agonist, further reinforcing adipogenic commitment. This mechanistic design underlies the consistency and robustness of the difAdipo model and supports its utility for EV studies in human adipose biology.

We utilized TFF for EV isolation, which offers distinct advantages for samples with high lipid content, such as adipocyte-derived EVs. TFF enables continuous diafiltration, thereby removing small soluble proteins and lipoproteins that frequently contaminate EV preparations obtained by ultracentrifugation or precipitation methods [52,53,54]. This strategy helps ensure a higher purity of EV isolates and minimizes potential confounding factors in downstream characterization. Furthermore, we confirmed efficient uptake of both difAdipo-EVs and mAdipo-EVs by HMC3 and THP-1 recipient cells. This initial evidence of EV internalization provides a foundation for our planned downstream functional assays. Adipocyte-derived EVs are known to transport a diverse array of bioactive cargo, including adipokines, metabolic enzymes, lipids, and regulatory non-coding RNAs. These molecular constituents have been shown to participate in intercellular signaling and influence systemic processes such as insulin sensitivity, immune modulation, and energy homeostasis [33,55,56]. Notably, several EV-associated miRNAs—including miR-27a and miR-155—play critical roles in modulating macrophage polarization and adipose tissue inflammation [57,58]. Including this context underscores the potential functional impact of difAdipo-EVs and mature adipocyte-EVs and provides a foundation for future mechanistic studies on EV-mediated adipose tissue communication. Due to recognized cross-species differences [26,59] between mouse and human adipose biology, adipocyte EVs from rodent models have limited direct human translational utility. Several alternate approaches to circumvent the cross-species differences include studying in vitro differentiated human adipocytes, adipose tissue explants, and circulating adipocyte-enriched EV subpopulations in human biofluids [60]. Kranendonk and colleagues were amongst the earliest groups to characterize human adipocyte EVs (defined as adiponectin and fatty-acid binding protein 4-positive EVs), isolated from the Human Simpson Golabi Behmel Syndrome (SGBS) cell line [33]. More recently, a study by Clement and collaborators isolated primary human adipocytes from dermolipectomy-derived subcutaneous adipose tissue and demonstrated the functional ability of isolated human adipocyte EVs to metabolically remodel melanoma cells into a more invasive phenotype [61]. Several human ADEV studies have utilized tissue explants for ADEVs rather than mature adipocytes [37,62]. Human adipose tissue explants have the advantage of overcoming the cross-species limitations of mouse models but confound the ability to investigate the potential pathobiological role of EVs shed solely from mature adipocytes [60], which are the most abundant and primary functional metabolic cells within visceral adipose tissue [35,63].

A less invasive and more feasible approach for studying human ADEVs involves interrogating the circulating pool of all EVs and attempting to enrich for EV subpopulations that contain ‘adipocyte-specific’ markers. Connolly and colleagues were the first to confirm human EVs containing adipose-enriched markers within the circulating pool of plasma-derived EVs from obese individuals [64]. In this study, the authors applied contemporaneous ISEV guidelines [65] for EV research and used complementary EV isolation and enrichment methods to attempt to account for non-vesicular co-isolates and non-adipose EV populations.

One of the formidable challenges in studying cell- and tissue-specific EVs, such as ADEVs, arises from their low abundance and high signal-to-noise ratio [60,66]. Adipose EVs are estimated to comprise >80% of tissue-derived EVs in human plasma and can be the primary source of circulating miRNAs; however, tissue-derived EVs comprise <0.2% of the circulating human plasma pool [57,67,68]. This limitation underscores the critical need for more advanced methods to study low-abundant, rare EV subpopulations. To address this limitation, we first produced a surrogate source of functional mature adipocytes in vitro and demonstrated their ability to shed EVs comparable in adipocyte-specific markers to EVs shed by adipocytes that matured in vivo.

To our knowledge, no studies have characterized the biophysical properties of human adipose-derived EVs in the pre- and post-adipogenic phases from paired human biospecimens. One study conducted a systematic phenotypic comparison between immature and mature mouse ADEVs [69]. In this study, the authors characterized the biophysical properties of pre- and post-adipogenic adipocyte-derived EVs from 3T3-L1 cells. Similar to the Connolly study, we found the highest expression of the preAdipo marker PREF-1 in the earliest stages of differentiation and the highest expression of the mAdipo marker *ADIPOQ* in the later stages of maturation. They also found increased secretion of small EVs in the early stages of differentiation, slightly increased CD9 and CD63 proteins via time-resolved fluorescence, and a higher proportion of phospholipids associated with cell signaling. Interestingly, the authors found EV-unique characteristics that differed from the 3T3-L1 parental cell, suggesting modifications that could impact EV-mediated intercellular communication [69]. Interestingly, while our bulk transcriptomic analysis showed a significant downregulation of *OCT4* expression in mAdipos compared to early-stage preAdipos, single-EV analysis revealed an increased *OCT4* signal in difAdipo-derived EVs. This apparent discrepancy may reflect selective packaging during terminal differentiation, whereby residual pluripotency-associated transcripts or proteins are enriched within extracellular vesicles. Such cargo selection highlights the fact that EV content does not always directly mirror cellular transcript levels and may represent functionally relevant regulatory subpopulations. This observation underscores the utility of single-EV profiling to uncover nuanced aspects of EV heterogeneity and intercellular signaling.

Finally, we applied single-EV multiplexing analytics to validate the adipocyte specificity of our difAdipo-EVs. The colocalization of EV surface proteins and internal cargo can increase sensitivity for detecting low-abundance EV subpopulations and can also enhance the biomarker utility of EVs in detecting earlier stages of disease [70,71]. Therefore, our single ADEV multiplexing platform holds great promise for investigating novel and robust, adipocyte-specific EVs in obesity-associated diseases.

While our study demonstrates that difAdipos recapitulate key morphological, transcriptional, and functional features of mature adipocytes, several limitations should be acknowledged. First, inherent donor-to-donor variability in adipose tissue characteristics may influence differentiation efficiency and EV content, although we minimized this by using standardized culture protocols. Second, despite the robust in vitro differentiation observed, the difAdipo model does not fully capture the complexity of the in vivo adipose microenvironment, including hypoxia, extracellular matrix dynamics, and immune cell interactions. Finally, in vivo biodistribution and functional assays of difAdipo-derived EVs remain ongoing and are critical next steps to fully validate the physiological relevance of this system.

Despite these limitations, our model offers significant clinical potential. The use of difAdipo-EVs provides a scalable, reproducible, human-based platform for biomarker discovery in obesity-related diseases and for studying adipose tissue dysfunction. Moreover, this model could be applied toward the development of engineered therapeutic EVs aimed at modulating metabolic, inflammatory, or fibrotic pathways, offering promising avenues for translational applications in obesity and associated comorbidities.

## 5. Conclusions

Our study presents a novel paradigm for advancing the translational application of human ADEVs by using human difAdipos as a surrogate for mature adipocytes, thereby augmenting the harvesting of viable human-derived, mature adipocyte EVs. The model established provides a high-yield, functionally equivalent source of EVs, facilitating more consistent and relevant research into the role of ADEVs in obesity and metabolic diseases. Our findings underscore the importance of adipocyte differentiation on EV content and function, revealing significant differences in biomarker profiles between preAdipo-EVs and difAdipo-EVs. This differentiation may impact the bioactive cargo within the EVs, influencing recipient cell behavior and contributing to metabolic complications associated with obesity.

Future research should explore the specific mechanisms by which difAdipo-EVs influence metabolic processes and their potential as biomarkers or therapeutic targets in obesity-related conditions. This study herein lays the groundwork for developing therapeutic strategies that modulate ADEVs’ effects, offering promising avenues for mitigating the adverse consequences of obesity on metabolic health and reducing the risk of associated diseases.

## Data Availability

All data generated or analyzed during this study are included in this manuscript and its Supplementary Information file. Further inquiries can be directed to the corresponding author.

## Supplementary Materials

The following supporting information can be downloaded at: https://www.mdpi.com/article/10.3390/cells14110757/s1. Figure S1: Full-size uncropped Western blot images corresponding to the cropped blots shown in Figure 5C; Table S1: Participant demographics; Table S2: Definition and source of adipose cells used in this study; Table S3: Media composition; Table S4: Primer list; Table S5: Molecular beacon list.
