# Supplementary material for: Human Differentiated Adipocytes as Surrogate Mature Adipocytes for Adipocyte-Derived Extracellular Vesicle Analysis
Source: Cells. 2025 May 22;14(11):757. doi: 10.3390/cells14110757 (PMC12153563; doi:10.3390/cells14110757)
Supplement: Supplementary file 1 [file cells-14-00757-s001.zip › cells-3595193-supplementary.pdf]

Human Differentiated Adipocytes as Surrogate Mature Adipocytes for Adipocyte-Derived Extracellular Vesicle Analysis

Mangesh Dattu Hade [1,†], Bradley L. Butsch [1,†], Paola Loreto Palacio [1], Kim Truc Nguyen [2], Dharti Shantaram [3], Sabrena F. Noria [4], Stacy A. Brethauer [4], Bradley J. Needleman [4], Willa Hsueh [3], Eduardo Reátegui [2] and Setty M. Magaña [1,*]

1 Department of Pediatrics, Center for Clinical and Translational Research, Nationwide Children's Hospital, Columbus, 43202 OH, USA; mangeshdattu.hade@nationwidechildrens.org (M.D.H.); bradleybutsch@gmail.com (B.L.B.); paolaloreto1703@gmail.com (P.L.P.)
2 Department of Chemical and Biomedical Engineering, The Ohio State University, Columbus, 43210 OH, USA; nguyenkim.1@osu.edu (K.T.N.); reategui.8@osu.edu (E.R.)
3 Diabetes and Metabolism Research Center, The Ohio State University, Columbus, 43210 OH, USA; dharti.shantaram@osumc.edu (D.S.); willa.hsueh@osumc.edu (W.H.)
4 Center for Minimally Invasive Surgery, Department of Surgery, Division of General and GI Surgery, The Ohio State University Wexner Medical Center, Columbus, 43210 OH, USA; sabrena.noria@osumc.edu (S.F.N.); stacy.brethauer@osumc.edu (S.A.B.); bradley.needleman@osumc.edu (B.J.N.)
* Correspondence: setty.magana@nationwidechildrens.org; Tel.: +1-614-722-4625; Fax: +1-614-722-4633
† These authors contributed equally to this work.

Table S1: Participants demographics.

ID	Age	Sex	Ethnicity	BMI (kg/m^2)
**1**	31	F	African American	49.51
**2**	21	F	Caucasian	42.64
**3**	42	F	Caucasian	38.42
**4**	32	F	Caucasian	40.22
**5**	31	F	Caucasian	47.12
**6**	43	F	Caucasian	34.93
**7**	60	F	Caucasian	38.58
**8**	35	F	African American	46.83
**9**	43	F	Caucasian	34.67
**10**	55	F	Caucasian	30.18

**11**	65	F	Caucasian	47.59

Table S2. Definition and source of adipose cells used in this study.

Abbreviation	Definition	Study Source	EV population
preAdipos	preadipocytes	Derived from the stromal vascular fraction<br>• Differentiated in vitro for 28 days under adipogenic induction and growth media	preAdipo-EVs
difAdipos	In vitro differentiated adipocytes (surrogate source for mature/native adipocytes)	preAdipos	difAdipo-EVs
mAdipos	In vivo (native) differentiated mature adipocytes	Derived from adipose tissue dissociation<br>• Cultured ex vivo for 24 hours	mAdipo-EVs

Table S3: Media Composition

**AIM1**	**Ingredients**	**Stock Concentration**	**Final Concentration**
	DMEM/low glucose		60%
	MCDB201 Media		40%
	FBS		2%
	Penicillin/Streptomycin (P/S)	100x	1x

	Dexamethasone (DXM)	10uM	1nM
	L-Ascorbic acid 2-phosphate (LAAP)	50mM	0.1mM
	ITS MIX	100X	1x
	Linoleic acid-Albumin	100X	1x
**AIM2**			
	DMEM/low glucose		60%
	MCDB201 Media		40%
	FBS (-20°C)		2%
	Penicillin/Streptomycin (P/S)	100x	1x
	Insulin (rec. human) (INS)	10mg/mL (water)	5ug/uL
	Indomethacin (IND)	30mM (MeOH)	50uM
	IBMX	5mM (MeOH)	0.5uM
	Dexamethasone (DXM)	2mg/mL (5mM, EtOH)	1uM
	T3	10uM	1nM

Table S4: Primer list

**Gene**	**Forward sequence**	**Reverse Sequence**
AdipoQ	GGT CTT ATT GGT CCT AAG GG	GTA GAA GAT CTT GGT AAA GCG
plin1	GCG GAA TTT GCT GCC AAC ACT C	AGA CTT CTG GGC TTG CTG GTG T
PPARG	AAA GAA GCC AAC ACT AAA CC	TGG TCA TTT CGT TAA AGG C
Oct4	GTT GAT CCT CGG ACC TGG CTA	GGT TGC CTC TCA CTC GGT TCT
Pref-1	CTT TCG GCC ACA GCA CCT AT	TGT CAT CCT CGC AGA ATC CAT
GATA3	AGG GAC GTC CTG TGC GAA CT	GGT CTG GAT GCC TTC CTT CTT CAT

Table S5: Molecular beacon list

Name	Sequence
PPARG-799 MB	+CTT +CTC /iCy3/+CT T+CT C+GG C+CT GTG GGC CGA GAA G/3BHQ_2/
PPARG-799_oligo	GGC GGA TGC CAC AGG CCG AGA AGG AGA
ADIPOQ-353 MB	+ACT +CCG /iCy3/+GT +TT+C A+CC GAT GTC TCC CAT CGG TGA AAC /3BHQ_2/
ADIPOQ -353_oligo	CTA AGG GAG ACA TCG GTG AAA CCG GAG TAC

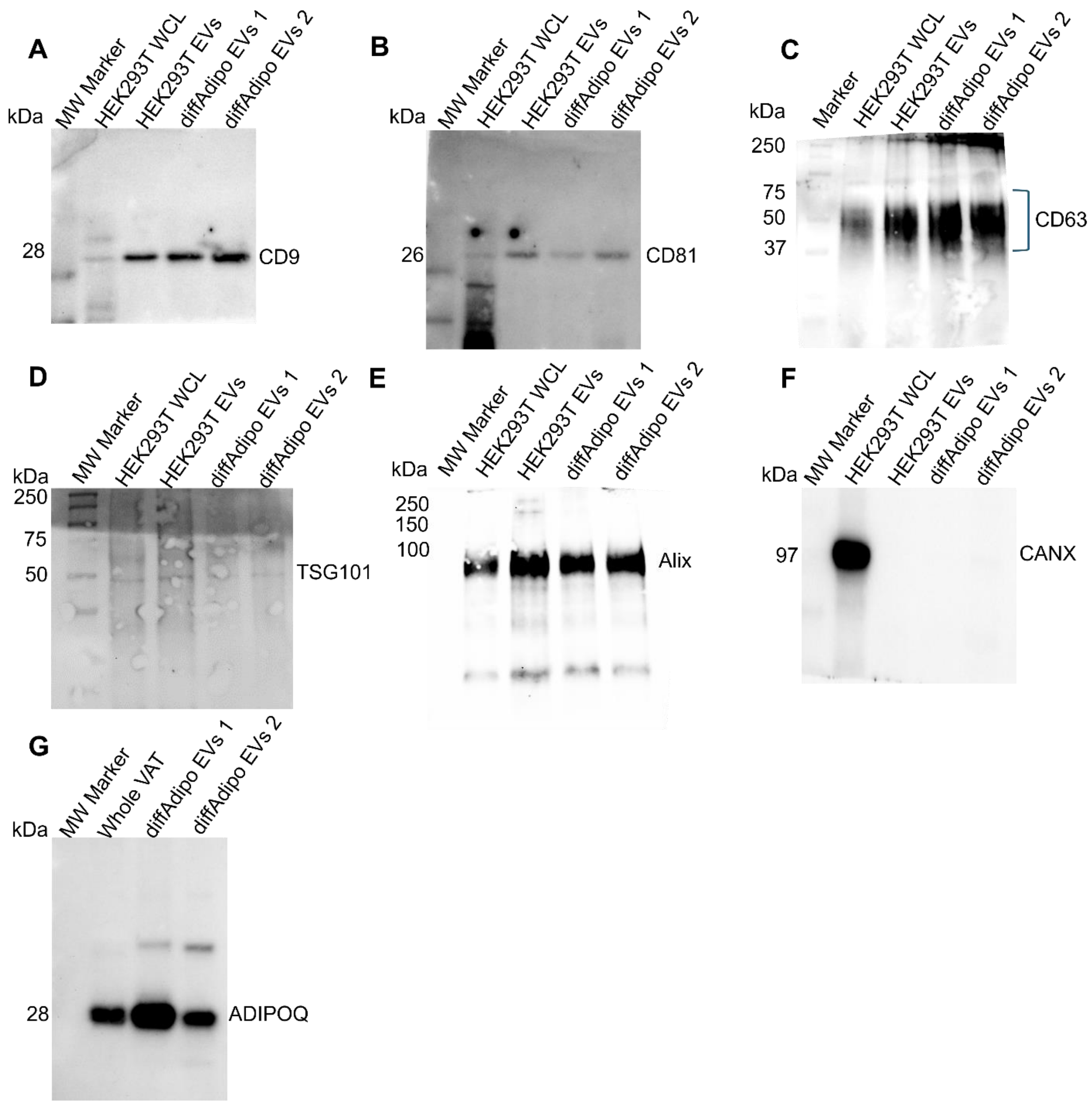

Supplementary Figure S1. Full-size uncropped Western blot images corresponding to the cropped blots shown in Figure 5C. EVs were isolated from differentiated adipocytes (difAdipo) of bariatric patients, referred to as EV1 (Patient 1) and EV2 (Patient 2). Each blot includes three lanes: molecular weight marker (left), difAdipo EV1, and difAdipo EV2. (A) Full-size blot for CD9 (~28 kDa), a common tetraspanin EV marker, showing positive expression in both difAdipo EV1 and difAdipo EV2. (B) Full-size blot for CD81 (~26 kDa), another EV-enriched tetraspanin, also detected in both samples. (C) Full-size blot for CD63 (~50 kDa), another EV-enriched tetraspanin, also detected in both samples. (D) Full-size blot for TSG101 (~47 kDa), another EV-enriched marker, also detected in both samples. (E) Full-size blot for Alix (~100 kDa), another EV-enriched marker, also detected in both samples. (F) Full-size blot for Calnexin (CANX; ~97 kDa), an endoplasmic reticulum protein used as a negative marker for EVs, which was absent in both difAdipo EV1 and difAdipo EV2, confirming minimal contamination by cellular debris. (G) Full-size blot for Adiponectin (ADIPOQ; ~28 kDa monomer and higher molecular weight oligomers), an adipocyte-specific protein, confirming the adipocyte origin of the difAdipo EVs. These full-size images are provided to validate the specificity, band integrity, and transparency of the immunoblotting data presented in the cropped panels of Figure 5C. Only minimal linear contrast adjustment was applied, and no other image modifications were made. WCL, whole cell lysate; VAT, visceral adipose tissue
